# Remyelination Therapy in Multiple Sclerosis

**DOI:** 10.3389/fneur.2015.00257

**Published:** 2015-12-10

**Authors:** Danielle E. Harlow, Justin M. Honce, Augusto A. Miravalle

**Affiliations:** ^1^Department of Cell and Developmental Biology, University of Colorado Anschutz Medical Campus, Aurora, CO, USA; ^2^Department of Radiology, University of Colorado Anschutz Medical Campus, Aurora, CO, USA; ^3^Department of Neurology, University of Colorado Anschutz Medical Campus, Aurora, CO, USA

**Keywords:** multiple sclerosis, remyelination, myelin repair, neuroprotection, MRI, MTI, DTI

## Abstract

Multiple sclerosis (MS) is an immune-mediated disorder of the central nervous system that results in destruction of the myelin sheath that surrounds axons and eventual neurodegeneration. Current treatments approved for the treatment of relapsing forms of MS target the aberrant immune response and successfully reduce the severity of attacks and frequency of relapses. Therapies are still needed that can repair damage particularly for the treatment of progressive forms of MS for which current therapies are relatively ineffective. Remyelination can restore neuronal function and prevent further neuronal loss and clinical disability. Recent advancements in our understanding of the molecular and cellular mechanisms regulating myelination, as well as the development of high-throughput screens to identify agents that enhance myelination, have lead to the identification of many potential remyelination therapies currently in preclinical and early clinical development. One problem that has plagued the development of treatments to promote remyelination is the difficulty in assessing remyelination in patients with current imaging techniques. Powerful new imaging technologies are making it easier to discern remyelination in patients, which is critical for the assessment of these new therapeutic strategies during clinical trials. This review will summarize what is currently known about remyelination failure in MS, strategies to overcome this failure, new therapeutic treatments in the pipeline for promoting remyelination in MS patients, and new imaging technologies for measuring remyelination in patients.

## Introduction

The therapeutic armamentarium for multiple sclerosis (MS) has expanded significantly in the last few decades due to better understanding of the basic pathophysiological mechanisms of the disease process. However, despite the development of increasingly effective therapies, a cure for MS has not been found and MS patients continue to suffer from chronic progressive disability. Currently, approved treatments for MS work by reducing immune system activity or blocking entry of immune cells into the central nervous system (CNS). Although these treatments can reduce relapse rates and severity of attacks, they do not repair immune-mediated damage to the myelin sheaths surrounding axons. Chronic demyelination leads to degeneration of axons and eventually loss of neurons. Neuronal loss correlates highly with clinical disability, highlighting the need for treatments that promote neuronal survival in both relapsing and progressive forms of MS. Experimental models of MS (both *in vitro* cultures and *in vivo* studies) have shown that preservation of myelin and remyelination of axons can increase neuronal survival (1, 2). To protect neurons, restore function, and halt the progression of disability, additional treatments need to be developed to promote myelin repair and neuronal protection. In this article, we will review current concepts of effective remyelination in MS including proposed mechanisms of myelin regulation, emerging therapies that might contribute to repair and restore cell function in MS, and the use of magnetic resonance imaging (MRI) to measure remyelination in clinical trials.

## Factors That Contribute to Remyelination Failure in MS

Understanding why endogenous remyelination often fails in MS is essential to the development of effective remyelination and repair strategies. Myelination of axons by oligodendrocytes in the CNS is a dynamic process determined by both the cytoarchitecture and microenvironment of the brain, spinal cord, and optic nerves. To ensure proper myelination of axons, oligodendrocytes regulate both their numbers and the amount of myelin each cell produces to properly match the number, diameter, and length of axons they encounter. After the completion of developmental myelination, many oligodendrocyte progenitor cells (OPCs) persist in the adult CNS. Unlike neurons, which fail to regenerate after CNS insult, adult OPCs are capable of proliferating and differentiating into mature oligodendrocytes that myelinate axons in response to injury or damage (3–5). Despite this regenerative ability, why do many axons remain demyelinated in the CNS of MS patients?

One hypothesis for remyelination failure is that the number of adult OPCs available for remyelination is depleted over time (1, 2, 4), however, post-mortem examinations of MS patients, including those in the seventh and eighth decades of life, revealed the presence of OPCs throughout the CNS, including within MS lesions (5–7). Nonetheless, many OPCs fail to mature into myelin-producing oligodendrocytes. These observations suggest that the lesion microenvironment is prohibitive to OPC differentiation and subsequent remyelination of axons.

Many changes occur in areas of demyelination that could prevent remyelination by endogenous OPCs [reviewed by Ref. (8, 9)]. Disruptions to the blood–brain barrier, basal lamina disturbances, and vasculature leakage occur (10–12). This leads to aberrant deposition of extracellular matrix (ECM) components, including fibronectin, hyaluronic acid (HA), and chondroitin sulfate proteoglycans (CSPGs), which can block the differentiation of OPCs and premyelinating oligodendrocytes (8, 9, 13–18). Demyelination can expose OPCs within lesions to other inhibitory cues, including components of damaged myelin such as the proteins MAG (myelin-associated glycoprotein), OMgp (oligodendrocyte myelin glycoprotein), and Nogo-A that signal through the Nogo receptor 1 and its co-receptors p75^NTR^, TROY, and LINGO-1 (leucine-rich repeat- and Ig domain-containing Nogo receptor-interacting protein 1) to inhibit both axonal regeneration as well as oligodendrocyte differentiation and remyelination (19–23). Activation of both innate CNS and peripheral immune cell populations leads to the release of soluble factors, such as pro-inflammatory cytokines, that can also negatively impact remyelination (24–27).

In particular, semaphorins, originally described as guidance molecules for axons, have been shown to play important roles in the regulation of remyelination. The soluble class III semaphorins (sema) 3A and 3F have been shown to be upregulated in active demyelinating but not chronic lesions in MS patients (28). These molecules are known to influence OPC migration, with sema 3A repelling OPCs and sema 3F attracting OPCs (29–31). Modulation of semaphorin levels within the lesion environment via viral overexpression impacts OPC migration and subsequent remyelination in animal models of demyelination suggests that relative levels of semaphorins in MS lesions may impact remyelination (31, 32). Interestingly, sema 3A and 3F are upregulated in the neuronal cell bodies of demyelinated axons far from lesions sites, raising the possibility that changes in demyelinated neurons may also influence their potential to be remyelinated (28). The transmembrane-bound semaphorin 4D (CD100) is normally expressed by mature MAG-expressing oligodendrocytes but not NG2-positive OPCs. Sema 4D is upregulated after injury and increases oligodendrocyte cell death (33, 34). Overexpression of sema 4D inhibits myelination in oligo-neuronal co-cultures while knock-down of sema 4D in a model of spinal cord injury (SCI) promotes functional recovery (35). Additionally, sema 4D expression disrupts endothelial tight junctions (35, 36). Recently, an antibody against sema 4D was used to treat animals in the experimental autoimmune encephalomyelitis (EAE) model and resulted in improved blood-brain barrier (BBB) integrity and improved OPC differentiation and axonal myelination (37). A phase I trial evaluating safety of a humanized anti-Sema4D IgG4 antibody in MS patients was recently completed (38).

As lesions develop, astrogliosis, a hallmark of MS pathology, occurs in and around lesions, resulting in both structural and biochemical changes. Astrocyte secretion of cytokines can influence lymphocyte infiltration (39, 40) while secretion of cytotoxic factors such as reactive oxygen and nitrogen species, glutamate, and ATP may directly affect oligodendrocytes and neurons (41–44). It is important to note that not all astrocyte changes are pathologic and some may even contribute to repair. For example, BDNF derived from astrocytes has been shown to play an important role in remyelination after cuprizone-induced demyelination (45). Astrocytes may also suppress inflammation and protect neurons from damaging reactive oxygen species in MS lesions via regulation of mitochondrial antioxidant enzymes (46). Clearly, the role of astrocytes in MS pathology is a complicated one and much remains to be elucidated (47, 48), but reduction of astrogliosis has beneficial effects on remyelination in several animal models of demyelination (49, 50).

Disruptions in the glial–neuronal network between astrocytes, oligodendrocytes, and neurons also lead to metabolic deficiencies across cell types that cause cellular dysfunction and death (51–56). As neurodegeneration proceeds, unhealthy axons may no longer be receptive to remyelination; therefore, strategies that improve neuronal survival may also lead to increased remyelination, and importantly, improved clinical outcomes.

Several non-disease-related factors such as age, sex, diet, and individual genetic background can also impact the efficiency of remyelination (57–61). For example, with age, remyelination occurs more slowly due to changes in the CNS environment and intrinsic epigenetic changes in oligodendrocytes, as well as age-related changes in the immune response (60, 62–66). Another interesting observation is that females are at higher risk of developing MS, yet may also remyelinate more efficiently than males, which could be due to the differential effects of sex hormones on oligodendrocyte proliferation and maturation as well as on the neuroinflammatory process (57, 58, 67, 68).

## Proposed Mechanisms of CNS Repair and Remyelination in the Context of MS

There are several potential strategies to enhance the remyelination capacity of endogenous OPCs, such as manipulating intrinsic signaling pathways within oligodendrocytes to override the inhibition of remyelination or altering the lesion environment to be more permissive of OPC differentiation and remyelination. Transplantation to increase OPC numbers is probably not necessary in MS given the large numbers of OPCs present in adult brain tissue. Likewise, if endogenous OPCs sense injury and enter lesions, but cannot differentiate there, then enhancing the overall proliferation/migration of OPCs is unlikely to dramatically alter their capacity to remyelinate axons. Therefore, after dampening inflammation, it is crucial to support the survival and differentiation of endogenous adult OPCs in order to stimulate remyelination within the altered microenvironment of MS lesions, while also prolonging survival of denuded axons so that they may be effectively remyelinated.

### Modulation of Intrinsic Signaling Pathways

An important approach to myelin preservation and repair is the pharmacological manipulation of intrinsic signaling pathways. Small molecules that can target specific components of the signaling pathways that underlie myelination will need to be developed and tested. Fortunately, much has been learned about the many signaling pathways that govern oligodendrocyte differentiation and myelination over the last three decades (see Table 1). Several groups have recently utilized small molecules capable of modulating these pathways to enhance myelination as discussed below. Complicating matters, however, there is a tremendous amount of cross-talk among signaling pathways, and manipulation of one pathway often induces alterations in another pathway (69). The high degree of cross-talk highlights the need for continued study of the interaction of these pathways in myelinating cells to aid therapeutic intervention.

**Table 1:** **Selected pathways and molecules that influence myelination**.

Signaling pathway	Impact on oligodendrocytes and myelination	Levels in MS	Reference
Notch	Spatial regulation of OPCs	In active MS lesions: Notch1 is expressed by non-differentiated oligodendrocytes, and Jagged1 is expressed by hypertrophic astrocytes. GWAS identified *Jagged1* as susceptibility gene for MS	(70–74)
Wnt	Negatively regulates production and differentiation of oligodendrocytes. Inhibition of Wnt via Axin2 promotes differentiation and myelination	Wnt signaling and proteins are elevated in active MS lesions	(75–78)
Akt-mTOR	Powerful positive regulator of myelination without dramatically impacting specification or proliferation of OPCs	Not determined	(79–83)
Erk1/2 MAPK	Regulates myelin thickness without impacting oligodendrocyte numbers, or specification, or differentiation	Not determined	(84–86)
RXR/PPAR	Stimulation of RXR/PPARs inhibits microglial activation and accelerates remyelination	RXRγ levels are high in active and remyelinating lesions and very low in chronic inactive lesions	(87–89)
ISR	Stress resistance and protection	ISR proteins CHOP, ATF4, and p-eIF2alpha are highly upregulated in MS lesions	(90–93)

*mTOR, mammalian target of rapamycin; RXR, retinoic acid receptors; PPAR, peroxisome proliferator-activated receptor; ISR, integrated stress response*.

### Altering the Extracellular Environment

Unquestionably, the extracellular environment is altered in MS lesions, and more effective remyelination would likely be achieved if the local environment within lesions could be restored (8, 9). To this end, clearance of myelin debris and the glial scar produced by astrocytes may enhance remyelination. Attempts to modify the ECM with enzymatic digestion, particularly CSPGs and HA, in the context of SCI have generated favorable results (94–96). This is possible due to the defined local area of damage in SCI that is easily identified and accessed for the application of ECM degrading enzymes. The challenge for such an approach in MS is the disparate and currently unpredictable pattern of demyelinated regions throughout the CNS, making local delivery difficult. Therefore, strategies that directly impact cells that produce ECM components, such as astrocytes, or alter oligodendrocyte responses to aberrant ECM molecules may be more successful approaches.

### Enhancement of Cell Survival

Disruptions to glial–neuronal networks and subsequent metabolic changes also impact OPCs and myelination. During acute demyelination, there is an increase in the mitochondrial content of axons early on in an attempt to keep up with the increased energy demands of denuded axons (97–99); however, over time this can lead to increased production of free radicals that can perpetuate axonal injury in the context of chronic demyelination (100). Providing metabolic support (e.g., lactate supplementation or reducing metabolic stress signaling) could preserve both neurons and oligodendroglia.

Finally, it is also important to note that acute inflammation is an important signal that activates adult OPCs to mobilize and mature, but long-term inflammation can be cytotoxic to OPCs (101, 102). Reducing the duration of astroglial and microglial activation could have several beneficial effects (e.g., reducing ECM deposition and decreasing pro-inflammatory cytokine production), which could be more conducive for OPC survival, differentiation, and remyelination. Another potential target for therapeutic intervention is to increase protection of oligodendrocytes in response to inflammation. Care must be taken, however, as the same signal can often have dual effects, either anti- or pro-inflammatory depending on the context. For example, silencing interferon-gamma (IFNγ) in astrocytes alleviates symptoms in EAE, whereas silencing of IFNγ in microglia increases disease severity (103).

## Remyelination Therapies in the Pipeline for the Treatment of MS

### Remyelination Therapies

Drugs that have a positive impact on remyelination and neuroprotection (Table 2) could be used as part of a combination therapy, including immunomodulatory drugs. Treatments that enhance the speed of remyelination are predicted to protect neurons from axonal degeneration. Intervention to maintain and repair myelin should occur early as chronically demyelinated axons will degenerate precluding future remyelination by existing OPCs and oligodendrocytes (104).

**Table 2:** **Potential remyelinating and neuroprotective therapies in multiple sclerosis**.

Drug	Proposed mechanism	Results	Reference
Anti-ASIC-1	Blockage of ASIC-1 prevents excessive intracellular accumulation of injurious Na(+) and Ca(2 +) in MS lesions	Clinical studies suggest neuroprotection as measured by brain atrophy during treatment compared with pretreatment.	(105, 106)
Anti-LINGO-1	Function-blocking anti-LINGO-1 antibodies enhance OPC differentiation and myelination	Phase 2 trial in patients with a first episode of optic neuritis showed an improvement on nerve impulse conduction along the affected optic nerve. Phase 2 trial in RRMS is ongoing.	(107, 108)
Benztropine	Antagonism of M1/M3 muscarinic acetylcholine receptors with subsequent stimulation of oligodendrocyte differentiation	In experimental models of MS, benztropine induced the differentiation of OPCs, and enhanced remyelination.	(109)
Guanabenz	α2 adrenergic receptor agonist. Protects oligodendrocytes by preventing dephosphorylation of eIF2, increasing oligodendrocyte survival and prevention of myelin loss.	Preclinical studies demonstrated improvement of deficits in EAE. Phase I clinical studies are ongoing.	(110, 111)
Laquinimod	Modified quinolone derivative; reduces microglia and astrocyte activation; increases neuroprotection and myelin preservation	Clinical studies suggest neuroprotection as measured by brain atrophy in treated versus untreated patients.	(112–117)
Miconazole and clobetasol	Activates eIF2, TX/RXR, and cholesterol signaling	Promoted oligodendrocyte differentiation and enhanced remyelination in *in vivo* models	(118)
Olesoxime	Decreases oxidative stress. Promotes oligodendrocyte maturation and myelin synthesis	Accelerated oligodendrocyte maturation and enhanced myelination *in vitro* and *in vivo* without affecting oligodendrocyte survival or proliferation. Phase 1 trail in MS patients completed.	(119, 120)
Quetiapine fumarate	Stimulates proliferation and maturation of oligodendrocytes, increases neurotrophic factors, and inhibits activated microglia, astrocytes, and T lymphocytes	Remyelinating and neuroprotective properties in EAE	(121, 122)
rHIgM22	rHIgM22 binds to the surface of oligodendrocytes promoting myelin repair	Preclinical studies indicate that it may protect oligodendrocytes and stimulate myelin repair. Phase I study demonstrated acceptable safety profile.	(123–125)

*OPC, oligodendrocyte precursor cells; eIF2, Eukaryotic Initiation Factor 2; CNS, Central Nervous System; LINGO, leucine-rich repeat and immunoglobulin-like domain-containing, Nogo receptor-interacting protein; rHIgM22, recombinant human IgM antibody 22; EAE, experimental autoimmune encephalomyelitis; ASIC-1, acid-sensing (proton gated) ion channel 1*.

High-throughput screens of previously FDA-approved drugs have identified several classes of drugs that enhance OPC differentiation and myelination. Although the beneficial effects of these drugs on CNS cells are encouraging, careful study of off-target effects will need to be undertaken, given that many of these drugs were originally utilized for non-CNS targets. Anticholinergics, including the drugs benztropine (109) and clemastine (126), were identified in two separate high-throughput screens looking for agents that enhance myelination. Activation of muscarinic receptors inhibits the differentiation of oligodendrocytes (127); therefore, the pro-myelinating effects of benztropine and clemastine are likely via their antagonism of M1/M3 muscarinic acetylcholine receptors. These anti-muscarinic compounds appear to enhance remyelination through direct effects on oligodendrocytes and not immunosuppressive effects. As phase 1 safety profile testing of anticholinergics has already been done for other indications, there has been a rapid translation of the preclinical laboratory findings to phase 2 clinical trials on the efficacy of clemastine in MS (128).

A third small molecule screen identified two additional FDA-approved drugs that promote oligodendrocyte differentiation and enhance remyelination in *in vivo* models (118): miconazole, an antifungal agent, and clobetasol, a corticosteroid used to treat eczema and psoriasis. Both act directly on oligodendrocytes as remyelinating drugs and impact eIF2 signaling, thyroid hormone receptor/retinoic acid receptor (TX/RXR) activation, and cholesterol signaling. Interestingly, miconazole acts through MAPK signaling and has no effect on the immune system, whereas clobetasol acts through glucocorticoid receptor signaling and is also a potent immunosuppressant in addition to being a remyelinating agent. With their high safety profile already established, approval for phase 2 trials to establish efficacy in MS could occur quickly.

Another strategy for promoting remyelination in MS is altering the local environment (soluble factors released by innate CNS cells, modifying the ECM), to be more permissive to endogenous OPCs. As mentioned above, the addition of enzymes, such as chondroitinase and hyaluronidase, is not very practical in a multifocal demyelinating disease such as MS. Therefore, an alternative approach is to change cellular responses to hostile environments. One promising target is the Nogo-A co-receptor LINGO-1, which is expressed by both neurons and oligodendrocytes. LINGO-1 acts as a negative regulator of oligodendrocyte differentiation and myelination. LINGO-1 knockout mice have enhanced remyelination in several demyelination models, including EAE and toxin-induced demyelinated lesions (23, 129, 130). Function-blocking anti-LINGO-1 antibodies enhance OPC differentiation and myelination (129). Promising results were obtained in a Phase 2 trial of a human IgG1 anti-LINGO-1 monoclonal antibody (BIIB033) in patients with a first episode of optic neuritis (107). Treatment was well tolerated; and after 24 weeks, those patients given the anti-LINGO antibody had faster nerve impulse conduction along the optic nerve than before treatment compared to those on placebo, indicating myelin repair. Although visual evoked potentials were increased, neither visual acuity nor retinal neuron layer thickness were improved. Another phase 2 trial in patients with relapsing MS is ongoing with estimated completion in June 2016 (108).

Preclinical studies of a recombinant human IgM antibody (rHIgM22) indicate that it may protect oligodendrocytes and stimulate myelin repair (123, 124). rHIgM22 binds to the surface of oligodendrocytes, and prevents their apoptosis. Although the exact target is not known, rHIgM22 may bind αvß3 integrins, the vitronectin/fibronectin receptor, and aggregated fibronectin has been shown to inhibit OPC differentiation and remyelination (131, 132). rHIgM22 also seems to inhibit OPC differentiation, so how it promotes myelin repair remains unclear. rHIgM22 was found safe and tolerable in Phase 1 clinical trials after a 6-month follow-up of a single dose of patients who remained on their existing MS treatments (133). A second dose-escalating phase 1 trial is underway (125).

Oligodendrocytes have very high metabolic demands due to production of extensive amounts of lipid-rich myelin membrane. This makes oligodendrocytes particularly sensitive to disruptions in metabolic homeostasis. Hypoxia/ischemia, oxygen–glucose deprivation, viral infections, and high rates of protein synthesis in the endoplasmic reticulum (ER) can all lead to ER stress, which activates pancreatic ER kinase (PERK) phosphorylation of eukaryotic translation initiation factor 2α (eIF2α) and the integrated stress response (ISR) (90). The ISR inhibits global protein synthesis to reduce the load on the ER and also upregulates transcription factors, such as STF4, CHOP, ATF4, and p-eIF2σ that cause the cell to be in a more stress-resistant state. Upregulation of these ISR targets is seen in MS lesions, with increased PERK activity in oligodendrocytes (91). Enhancement of the ISR in oligodendrocytes could protect them and lead to increased remyelination efficiency.

Guanabenz is an α2 adrenergic receptor agonist that has been previously approved by the FDA for the treatment of hypertension (134). Guanabenz alleviates symptoms in EAE, increases oligodendrocyte survival, and reduces CD4+ T cell accumulation in the CNS (110). Guanabenz appears to work by protecting oligodendrocytes against the inflammatory CNS environment. Guanabenz enhances the protective ISR of oligodendrocytes by preventing dephosphorylation of eIF2α, increasing oligodendrocyte survival, and protecting against myelin loss (110, 135). A phase 1 safety trail of oral guanabenz in MS patients recently began recruiting patients (111).

Olesoxime, is a cholesterol-oxime compound and mitochondrial pore modulator, originally developed to treat amyotrophic lateral sclerosis that targets proteins of the outer mitochondrial membrane, preventing permeability and decreasing oxidative stress (136). Olesoxime accelerates oligodendrocyte maturation and enhanced myelination *in vitro* and *in vivo* without affecting oligodendrocyte survival or proliferation (119). A phase 1b study in MS is ongoing (120).

Stem cell-based remyelinating therapies are also a plausible alternative strategy in MS as stem cells of both neural and mesenchymal origin have the ability to facilitate endogenous reparative processes, participate directly in remyelination, and attenuate neuroinflammation. However, there are crucial questions that have to be addressed before considering clinical studies, including the determination of the optimal cellular platform, the route of cell delivery, and candidate patients for treatment (137).

### Neuroprotective Strategies

In order to achieve maximal levels of remyelination, therapies that decrease axonal degeneration and increase neuronal survival in response to demyelination could be used to extend the period during which axons could be remyelinated.

Quetiapine fumarate is an atypical antipsychotic that has been shown to have both remyelinating and neuroprotective properties in EAE (121). It appears to impact various biological pathways relevant in MS with potential to stimulate proliferation and maturation of oligodendrocytes, release of neurotrophic factors, and inhibit activated microglia, astrocytes, and T lymphocytes (121). A dose-finding trial in both relapsing and progressive forms of MS is underway (122).

The acid-sensing ion channel, ASIC-1, contributes to the excessive intracellular accumulation of injurious Na(+) and Ca(2+) and is over-expressed in acute MS lesions. Blockade of ASIC1 through amiloride, a potassium-sparing diuretic that is currently licensed for hypertension and congestive cardiac failure, showed neuroprotective and myeloprotective effects in experimental models of MS (105, 106). A pilot study in primary progressive MS showed positive effects on brain volume (105) and a multi-arm randomized Phase II trial in secondary progressive MS patients is currently enrolling (138).

Promising results have been achieved with laquinimod, a small molecule that in addition to its effects on lymphocytes can also reduce glial reactivity (139, 140). Although the exact mechanism of action is not known, laquinimod, a modified quinoline derivative, appears to have direct effects on astrocytes and microglia/macrophages within the CNS (49, 117, 139, 140). This modulation of astrocyte and microglia responses appears to provide a protective effect on both neuronal function and oligodendrocytes and myelin within lesion areas (112, 141, 142). In phase 3 clinical trials of relapsing-remitting MS patients, both clinical disability and brain atrophy were reduced with laquinimod treatment indicative of a neuroprotective effect (113, 114, 143). A phase 2 trial for laquinimod in primary progressive MS is underway (115).

## Assessment of Remyelination in Clinical Studies

One of the biggest difficulties in the development of remyelination therapies for MS is the demonstration of remyelination in living patients. Indirect measures, such as improvements in neurophysiological outcomes, such as electroencephalography, evoked potentials, optical coherence tomography, and transcranial magnetic stimulation may suggest, yet not confirm, remyelination (144–148). Functional improvements could indicate remyelination, but could also indicate neuronal plasticity or the spreading of sodium channels into demyelinated internodes, which could also restore conduction in unmyelinated axons (144, 149, 150). Although an extensive discussion is beyond the scope of this review, analysis of serum and cerebrospinal fluid (CSF) biomarkers could provide important and specific information regarding remyelination and repair in the future. At present, candidate biomarkers appear to relate to disease activity (interleukin-6 or its soluble receptor, nitric oxide and nitric oxide synthase, osteopontin, and fetuin-A) or neurodegeneration and blood–brain barrier dysfunction (neurofilaments, tau, 14-3-3 proteins, S-100β, GFAP), rather than repair [for review, see in Ref. (151, 152)]. Even post-mortem, histopathologic approaches to detect remyelination are inherently limited to a “snapshot” assessment of the fluctuating process of demyelination and remyelination (153).

Demyelinating lesions are readily detected by T2-weighted (T2W) and fluid attenuated inversion recovery (FLAIR) MRI (154–156). Unfortunately, these sequences are unable to differentiate remyelination from the ever-changing milieu of local inflammation, edema, and axonal and myelin damage (153, 157–159). In addition, the correlation between MRI-identified lesion load and clinical disability is weak, a phenomenon referred to as the “clinical-radiologic paradox” in MS (160). With the increasing interest in development of new therapies for MS targeting remyelination, accurate MRI measures of myelin repair are crucial for assessing the impact of these therapies (161). Advancements in MRI technology may provide an opportunity for *in vivo* assessment of this dynamic process in both visible lesions and the putatively “normal appearing white matter (NAWM).” Candidate imaging sequences must be sensitive and specific for changes occurring *in vivo*, correlate with other indicators of remyelination, and crucially for quantitative MRI, must be reproducible across imaging platforms.

## Assessment of Remyelination by Advanced Imaging

### Diffusion Tensor Imaging

Diffusion tensor imaging (DTI) tracks the Brownian motion of water molecules in tissue to provide quantitative data about tissue microstructure, and is particularly well-suited to highly ordered environments such as CNS white matter (162). For different aspects of white matter microstructure, DTI produces various quantitative indices, including fractional anisotropy, mean diffusivity, axial diffusivity, and radial diffusivity. Fractional anisotropy reflects the overall vector of diffusion of water molecules and, by inference, the directionality of the fiber tracts, while mean diffusivity represents the overall magnitude of diffusion, regardless of direction (162). Both mean diffusivity and fractional anisotropy correlate with the number of axons and the degree of myelination in post-mortem examination, but it is difficult, if not impossible, to distinguish remyelination from demyelination using these metrics (163, 164). In highly ordered white matter bundles, radial diffusivity is thought to represent the diffusion of water molecules perpendicular to fiber tracts, while axial diffusivity is thought to represent the diffusion of water molecules parallel to the tract, and it has been suggested that radial diffusivity might be more sensitive to myelin damage while axial diffusivity may be more sensitive to axonal injury (164–167). While this appears promising, the major limitation of the radial and axial diffusivity indices is that the models used for derivation depend on accurate calculation of the correct fiber tract orientation. Crossing fibers and disruption of the white matter microstructure by varying pathology in MS (pro-inflammatory cellular infiltration, edema, demyelination, and axonal loss) may result in inaccurate determination of the diffusion tensor and underestimation of radial diffusivity, leading to underestimation of the degree of changes in myelination (168–171). Newer techniques such as high angular resolution diffusion imaging, which is capable of resolving crossing fibers (172), and neurite orientation dispersion and density imaging, which is more specific for myelination than standard DTI indices (173), may increase the specificity of these more advanced techniques to serve as biomarkers for tracking remyelination in MS.

### Magnetization Transfer Imaging

Magnetization transfer (MT) imaging exploits the exchange of magnetization that occurs between two “pools” of protons *in vivo*: a “free” pool of protons that are generally unbound in an aqueous environment, and a “restricted” pool of protons that are bound to high molecular weight molecules such as lipids and proteins (174, 175). While signal on conventional imaging is predominantly derived from the relaxation of protons in the “free” pool; in MT imaging, two sets of images are obtained (one with MT-weighting, and the other without) allowing imaging of both pools. An estimate of the magnetization transfer ratio (MTR) between these two pools can be calculated allowing for discrimination of subtle changes not picked up by conventional MRI.

**Figure 1:**
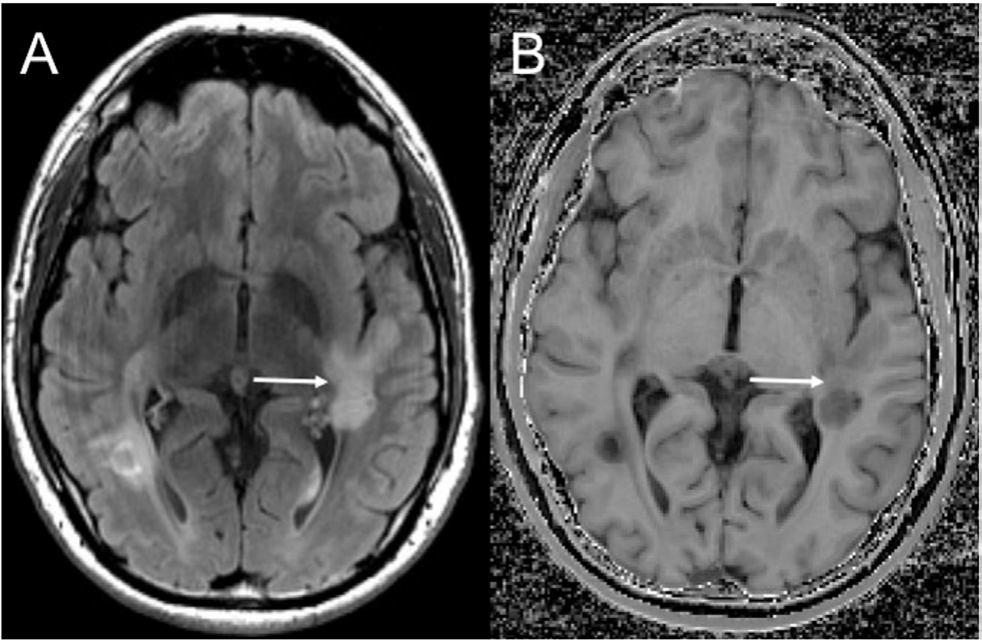
**Magnetization transfer imaging**. Axial FLAIR **(A)** demonstrating a large demyelinating lesion in the white matter posterior to the Sylvian fissure. Magnetization transfer image **(B)** demonstrates a band of normal white matter signal intensity across the mid aspect of the lesion (solid arrow) compatible with partial remyelination.

Although edema, inflammation, and axonal density do influence MTR, the high lipid content of myelin strongly affects the MTR (170, 176–178), allowing MT imaging to be relatively sensitive in detecting changes in myelination, including remyelination (Figure 1). White matter lesions have a lower MTR than NAWM, and NAWM in MS patients has a lower MTR than NAWM in controls (179–181). There is also variation between disease subtypes: secondary progressive MS has the lowest white matter MTR measures, followed by relapsing-remitting MS, and clinically isolated syndrome (CIS) (181). Remyelinated lesions have higher MTR than unmyelinated lesions, but MTR in remyelinated lesions remains lower than NAWM (153, 182–184), suggesting either that remyelination is incomplete or that newly formed myelin in lesions has a different structure, which is consistent with reports of thinner myelin in post-mortem examination of remyelinated lesions (185). While lesions appear generally static on conventional imaging over time, MTR fluctuates within lesions, suggesting alternating waves of both demyelination and remyelination (184). Given the semi-quantitative nature of MTR measurement, recent efforts have been made to directly measure the size and relaxation characteristics of the “restricted” proton pool, to provide quantitative measurements that are even more strongly influenced by myelin in the brain (186–190). Although performing multiple MT-weighted acquisitions increases scan times, MT imaging could be advantageous in large clinical trials as quantitative metrics are more consistent across scanners.

### Myelin Water Fraction Imaging

Brain tissue microstructure is complex and each tissue component displays different T2 relaxation characteristics. The differences in T2 relaxation correlate with water separated into three components: a long T2 component corresponding to CSF, an intermediate T2 component corresponding to intra/extracellular water, and a short T2 relaxation time component corresponding to water trapped with layers of myelin (191). Of most interest in MS is the short T2 (myelin water) component, measured as the ratio of myelin water to the total water, i.e., the myelin water fraction (MWF). Histopathological studies have shown that the MWF correlates with myelin content, is insensitive to changes related to inflammation, and is independent of axonal loss/degeneration (186, 192).

Early techniques for measuring MWF depended on 2D multi-spin echo acquisitions, which due to time considerations permitted only incomplete coverage of the brain. To improve brain coverage and further reduce MT effects, other sequences are now available, including 3D-GRASE and most recently mcDESPOT, which allow coverage of the entire brain within reasonable acquisition times (157, 193, 194). While these techniques show much promise, work still needs to be done to confirm their tissue specificity. One recent study, for instance, indicated that in white matter mcDESPOT may not be able to precisely estimate the two-pool model with exchange (195). Despite that limitation, MWF is lower in MS patients compared to that of controls (196), correlates with disability, and decreases over time in patients with progressive MS (197). MWF imaging has the potential to follow remyelination in lesions after acute edema has resolved (198) and has been shown to increase in patients treated with alemtuzumab, suggesting it may be useful in monitoring for remyelination in treated patients (199).

### Positron Emission Tomography

Positron emission tomography (PET) uses radioisotopes that directly bind to different tissue substrates to enable molecular imaging. Direct binding of imaging biomarkers specific for myelin could potentially far exceed the specificity of the other modalities discussed above and allow investigators to detect changes specific for myelin content in the brain. One such biomarker is the thioflavine-T derivative 2-(4′-methylaminophenyl)-6-hydroxybenzothiazole (PIB). ^11^C-PIB is currently used in the imaging of Alzheimer’s disease (200, 201) but recent studies have shown that in addition to amyloid plaques, PIB also has an affinity for CNS myelin and demonstrates differential binding to normal and demyelinated white matter (202, 203). In two RRMS patients, ^11^C-PIB uptake was less in enhancing lesions (203), indicating that there may be differences in the myelination of lesions at different stages. While promising, additional studies of more MS patients are required to validate these findings and not all centers will have access to such technology.

## Conclusion

Repair and remyelination in MS is possible, but remyelination often fails as a consequence of failure to recruit OPCs fully into the lesions, failure of OPCs to generate mature myelinating oligodendrocytes, and failure of oligodendrocytes to remyelinate axons. Several candidates to enhance remyelination are currently under investigation with a variety of mechanisms of action. One barrier to evaluating remyelination therapies in patients is the lack of methods to accurately detect myelination, demyelination, and remyelination with standard imaging technologies. Advancements in MRI technology allow better detection of myelin-specific changes but criteria that can be readily quantified using new imaging technologies will have to be established and validated to determine the success of remyelination in clinical trials.

## Conflict of Interest Statement

The authors declare that the research was conducted in the absence of any commercial or financial relationships that could be construed as a potential conflict of interest.
